# Gait Analysis Methods: An Overview of Wearable and Non-Wearable Systems, Highlighting Clinical Applications

**DOI:** 10.3390/s140203362

**Published:** 2014-02-19

**Authors:** Alvaro Muro-de-la-Herran, Begonya Garcia-Zapirain, Amaia Mendez-Zorrilla

**Affiliations:** DeustoTech-Life Unit, DeustoTech Institute of Technology, University of Deusto, Bilbao 48007, Spain; E-Mails: alvaro.muro@deusto.es (A.M.-H.); amaia.mendez@deusto.es (A.M.-Z.)

**Keywords:** gait analysis, wearable sensors, clinical application, sensor technology, gait disorder

## Abstract

This article presents a review of the methods used in recognition and analysis of the human gait from three different approaches: image processing, floor sensors and sensors placed on the body. Progress in new technologies has led the development of a series of devices and techniques which allow for objective evaluation, making measurements more efficient and effective and providing specialists with reliable information. Firstly, an introduction of the key gait parameters and semi-subjective methods is presented. Secondly, technologies and studies on the different objective methods are reviewed. Finally, based on the latest research, the characteristics of each method are discussed. 40% of the reviewed articles published in late 2012 and 2013 were related to non-wearable systems, 37.5% presented inertial sensor-based systems, and the remaining 22.5% corresponded to other wearable systems. An increasing number of research works demonstrate that various parameters such as precision, conformability, usability or transportability have indicated that the portable systems based on body sensors are promising methods for gait analysis.

## 1. Introduction

Analysis of the human gait is the subject of many research projects at the present time. A search on the Web of Knowledge for scientific articles that include “gait” in the title shows more than 3,400 publications between 2012 and 2013. Since research on this type of analysis was first begun in the 19th century, it has centered on achieving quantitative objective measurement of the different parameters that characterize gait in order to apply them to various fields such as sports [1–3], identification of people for security purposes [4–7], and medicine [8–10].

If we centre on the medical field, changes in gait reveal key information about persons’ quality of life. This is of special interest when searching for reliable information on the evolution of different diseases: (a) neurological diseases such as multiple sclerosis or Parkinson's; (b) systemic diseases such as cardiopathies (in which gait is clearly affected); (c) alterations in deambulation dynamic due to sequelae from stroke and (d) diseases caused by ageing, which affect a large percentage of the population. Accurate reliable knowledge of gait characteristics at a given time, and even more importantly, monitoring and evaluating them over time, will enable early diagnosis of diseases and their complications and help to find the best treatment.

The traditional scales used to analyse gait parameters in clinical conditions are semi-subjective, carried out by specialists who observe the quality of a patient's gait by making him/her walk. This is sometimes followed by a survey in which the patient is asked to give a subjective evaluation of the quality of his/her gait. The disadvantage of these methods is that they give subjective measurements, particularly concerning accuracy and precision, which have a negative effect on the diagnosis, follow-up and treatment of the pathologies.

In contrast to this background, progress in new technologies has given rise to devices and techniques which allow an objective evaluation of different gait parameters, resulting in more efficient measurement and providing specialists with a large amount of reliable information on patients’ gaits. This reduces the error margin caused by subjective techniques.

These technological devices used to study the human gait can be classified according to two different approaches: those based on non-wearable sensors (NWS) or on wearable sensors (WS). NWS systems require the use of controlled research facilities where the sensors are located and capture data on the gait while the subject walks on a clearly marked walkway. In contrast, WS systems make it possible to analyse data outside the laboratory and capture information about the human gait during the person's everyday activities. There is also a third group of hybrid systems that use a combination of both methods.

NWS systems can be classified into two subgroups: (1) those based on image processing (IP); and (2) those based on floor sensors (FS). IP systems capture data on the subject's gait through one or more optic sensors and take objective measurements of the different parameters through digital image processing. Analog or digital cameras are the mostly commonly used devices. Other types of optic sensors such as laser range scanners (LRS), infrared sensors and Time-of-Flight (ToF) cameras are also used. There are two systems within this category: with and without markers. The FS systems are based on sensors located along the floor on the so called “force platforms” where the gait information is measured through pressure sensors and ground reaction force sensors (GRF) which measure the force exerted by the subject's feet on the floor when he/she walks.

The WS systems use sensors located on several parts of the body, such as feet, knees, thighs or waist. Different types of sensors are used to capture the various signals that characterise the human gait. These include accelerometers, gyroscopic sensors, magnetometers, force sensors, extensometers, goniometers, active markers, electromyography, *etc.*

The main purpose of this paper is to review the latest advances in technologies and methods used to analyse the human gait, with particular emphasis in the field of medicine. Section 2 is divided into two subsections: (1) a description of the parameters that characterize the human gait and (2) a review of the semi-subjective techniques traditionally used in clinics. Section 3 offers a review of the objective techniques and methods that use sensors to measure the parameters of the human gait, showing the results of the most recent research. Section 4 includes a discussion and comparison of the latest advances and describes future research areas and lastly, Section 5 presents our conclusions.

## 2. Background to Gait Parameters

### 2.1. Parameters of Interest for the Human Gait

Research on the human gait comprises the qualitative and quantitative evaluation of the various factors that characterize it. Depending on the field of research, the factors of interest vary (see Table 1). For instance, for security purposes, interest may centre on distinguishing and identifying persons based on a general characterization of their silhouette and the movements between the subject's different body segments when walking [11]. However, in the field of sports, research may centre on analysis of the different forces exerted on each muscle through EMG [12]. From the clinical point of view, the importance of human gait analysis lies in the fact that gait disorders affect a high percentage of the world's population and are key problems in neurodegenerative diseases such as multiple sclerosis, amyotrophic lateral sclerosis or Parkinson's disease, as well as in many others such as myelopathies, spinal amyotrophy, cerebellar ataxia, brain tumours, craneoencephalic trauma, neuromuscular diseases (myopathies), cerebrovascular pathologies, certain types of dementia, heart disease or physiological ageing. Study of human gait characteristics may be useful for clinical applications, it has been the subject of numerous studies such as Mummolo *et al.*'s recent work [13] and may benefit the various groups suffering from gait-related disorders. There are studies on the elderly which link changes in various gait characteristics to gait deficiency [14]. The first symptoms of some neurological diseases are poor balance, a significantly slower pace, with a stage showing support on both feet [15]. Multiple sclerosis patients also show several gait alterations such as a shorter steps, lower free speed when walking and higher cadence than subjects without MS. In these cases, the knee and ankle joint rotation are distinctive for lower than normal excursion with less vertical ascent from the centre of gravity and more than normal bending of the trunk [16]. Another condition related to gait and balance deficiencies is osteoporosis [17], a systemic disease characterized by lower bone mass and deteriorated bone microarchitecture, which means more fragile bones and greater risk of fractures. In the elderly, physical exercise has a major impact on osteoporosis because it significantly helps to prevent falls, which are the biggest risk factor for this age group [18]. This condition is asymptomatic and may not be noticed for many years until it is detected following a fracture. Therefore, evaluation of gait quality may be valuable for early diagnosis.

Staff and medical associations working in the field of neurological diseases (and others) stress the need for constant control in high risk patients. This is currently done by subjective analyses of gait quality that only offer biased evaluations taken over short periods of time. These simple tests are not enough to give a reliable diagnosis because they only indicate the patients’ condition when they are being attended in the surgery and do not take into account their mobility throughout the day, week, month or longer term.

Accurate reliable knowledge of gait characteristics at a given moment, and more importantly, over time, will make early diagnosis of diseases and their complications possible, enabling medical staff to find the most suitable treatment. For example, gait velocity is a simple effective test that can identify subgroups of elderly patients who run a higher risk of death and severe morbidity following heart surgery [19]. Research projects such as sMartxa-basic, conducted in the Basque Country, Aragon and Languedoc-Roussillon, study the gait-related habits in the elderly in rural areas by long-term monitoring and analysis of the routes they take, distances and the uneven terrain they cover.

At the same time, many neurodegenerative and age-related diseases such as Parkinson's are linked to other parameters which make it possible to diagnose and know the patient's evolution. Some of these symptoms are altered balance and falls, agitation, tremors and changes in routine movements, *etc.*

Specialists assess patients’ health by using various methods that measure the parameters which most clearly represent the human gait. These are described below:

- Velocity
- Short step length (linear distance between two successive placements of the same foot)
- Long step or stride length (linear distance between the placements of both feet)
- Cadence or rhythm (number of steps per time unit)
- Step width (linear distance between two equivalent points of both feet)
- Step angle (direction of the foot during the step)
- Short step time
- Swing time for each foot (time from the moment the foot lifts from the floor until it touches it again, for each foot)
- Support time (time from the moment the heel touches the floor until the toes are lifted, for each foot)
- Distances travelled
- Gait autonomy (the maximum time a person can walk, taking into account the number and duration of the stops)
- Duration of the stops
- Existence of tremors when walking
- Record of falls
- Uneven terrain covered (height difference between drops and rises)
- Routes taken
- Gait phases
- Direction of leg segments
- Ground Reaction Forces
- Angles of the different joints (ankle, knee, hip)
- Electrical activity produced by muscles (EMG)
- Momentum and forces
- Body posture (bending, symmetry)
- Maintaining gait over long time periods

The parameters described above can be measured by two techniques to carry out an analysis that makes it possible to evaluate a person's health: (1) semi-subjective analysis techniques and (2) objective analysis techniques. The following section describes the semi-subjective techniques and Section 3 discusses the objective techniques.

### 2.2. Semi-Subjective Analysis Techniques

Semi-subjective methods usually consist of analyses carried out in clinical conditions by a specialist. The patient's various gait-related parameters are observed and evaluated while he/she walks on a pre-determined circuit. The following comprise a selection of the most common semi-subjective analysis techniques which are based on a medical specialist's observation of the patient's gait.

#### 2.2.1. Timed 25-Foot Walk (T25-FW)

This technique is known as the 25 foot walk test. This is the first part of the Multiple Sclerosis Functional Composite (MSFC), a standardised quantitative evaluation instrument consisting of three parts for use in clinical studies, particularly clinical tests on multiple sclerosis [20]. In this test, the specialist measures the time it takes the subject to walk a distance of 7 and a half meters in a straight line.

#### 2.2.2. Multiple Sclerosis Walking Scale (MSWS-12)

This scale assesses 12 parameters, taken from interviews with 30 patients, expert opinions and literature reviews which describe the impact of multiple sclerosis on patients’ gait [21]. However, because other neurological conditions affect motor skills, this test was later adapted to become a generic profile called Walk-12 [22].

#### 2.2.3. Tinetti Performance-Oriented Mobility Assessment (POMA)

In this test, the patient is required to walk forward at least 3 m, turn 180° and then walk quickly back to the chair. Patients should use their habitual aid (walking stick or walker) [23]. In a more recent study, Tinetti presented a reduced scale consisting of seven parameters according to two levels (normal or abnormal) that seem to accurately reflect the risk of falls. In the full version of the test, the section on balance disorders is based on 13 parameters organized in three levels and the study of the human gait is based on nine additional parameters classified in four levels. In conclusion, this test makes it possible to accurately evaluate elderly persons’ balance and gait disorders in everyday situations. However, the test requires a great deal of time with active participation from the subjects.

#### 2.2.4. Timed Get up and Go (TUG)

The TUG test is a timed test that requires patients to get up from a sitting position, walk a short distance, turn around, walk back to the chair and sit down again [24].

#### 2.2.5. Gait Abnormality Rating Scale (GARS)

This is a video-based analysis of 16 human gait characteristics. The GARS includes five general categories, four categories for the lower limbs and seven for the trunk, head and upper limbs [25].

#### 2.2.6. Extra-Laboratory Gait Assessment Method (ELGAM)

ELGAM is a method to evaluate gait in the home or community [26]. The parameters studied include step length, speed, initial gait style, ability to turn the head while walking and static balance. Low speed (under 0.5 m/s), short steps, difficulty turning the head and lack of balance are significantly linked to unstable gait.

## 3. Survey of Objective Techniques Used for Gait Measuring

In contrast to the semi-subjective techniques, objective gait analysis techniques are based on the use of different devices to capture and measure information related to the various gait parameters. These methods can be divided into three categories: those based on image processing (IP), on floor sensors (FS) and on sensors located on the body, carried by the users (wearable sensors—WS).There are a great many studies that demonstrate the validity of these sensors when quantifying and analysing the different aspects of the human gait. The following section contains an in-depth description of some studies on the newest technologies used in human gait analysis and recognition. They are organised according to the three categories described above.

### 3.1. Image Processing

The typical IP system is formed by several digital or analog cameras with lens that can be used to gather gait-related information. Techniques such as threshold filtering which converts images into black and white, the pixel count to calculate the number of light or dark pixels, or background segmentation which simply removes the background of the image, are just some of the possible ways to gather data to measure the gait variables. This method has been widely studied in order to identify people by the way they walk [27–29]. In the medical diagnosis field, Arias-Enriquez *et al.* presented a fuzzy system able to provide a linguistic interpretation of the kinematic analysis for the thigh and knee [30]. Recent research shows promising results on gait recognition by taking into account changes in the subject's path [31]. In [32], Muramatsu *et al.* solve the problem of decreased recognition accuracy due to the different views of the compared gallery and probe, applying a gait-based authentication method that uses an arbitrary view transformation scheme.

**Figure 1.**
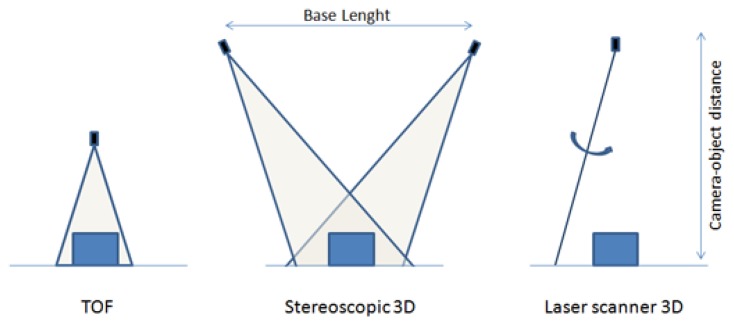
Different technologies for IP based measurement. Reproduced with permission from MESA Imaging.

Within IP methods, one technique has become very important at the present time: depth measurement, also called range imaging. This is a collection of techniques used to calculate and obtain a map of distances from a viewpoint [33]. These techniques make it possible to obtain important elements of the image with a better and faster real-time process. There are several technologies that can be applied for this purpose (Figure 1), such as camera triangulation (stereoscopic vision), laser range scanner [34], and Time-of-Flight methods [35]. Other studies use structured light [36,37], and infrared thermography [38].

#### 3.1.1. Stereoscopic Vision

This method can be used to determine the depth of points in the scene, for example, from the midpoint of the line between their focal points. In order to solve the problem of depth measurement using a stereo camera system, it is necessary to first find corresponding points in different images. This technique is based on the creation of a model through the calculation of similar triangles between the optical sensor, the light-emitter and the object in the scene. Creating a camera model involves acquiring multiple images, usually of a calibration grid, in multiple planes. This technique is widely used for gait analysis [11,39].

#### 3.1.2. Time-of-Flight Systems (ToF)

**Figure 2.**
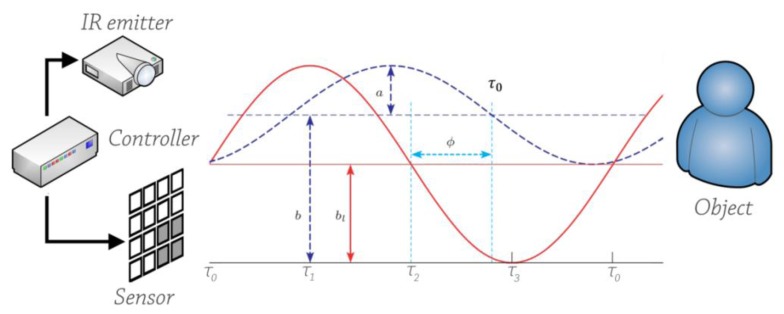
Time-of-flight working principle.

ToF systems are based on cameras using signal modulation that measure distances based on the phase-shift principle [40] (Figure 2). The observed scene is illuminated with modulated near infrared light (NIL), whereby the modulation signal is assumed to be sinusoidal with frequencies in the order of some megahertz. The reflected light is projected onto a charge coupled device (CCD) or complementary metal oxide semiconductor (CMOS) sensor or a combined technology. There, the phase shift, which is proportional with the covered distance, is measured in parallel within each pixel. Let S_i_(t) = {s_i_(t_0_),s_i_(t_1_),…,s_i_(t_m_)|i = 1,…,n}) be m + 1 measurements of an optical input signal taken at each of n pixel locations in the image array. Further let A = ({a_i_|i = 1,…,n}) be the set of amplitude data and B = ({b_i_|i = 1,…,n}) the set of intensity (offset) data. From the reflected sinusoidal light four measurementss_i_(τ_0_), s_i_(τ_1_), s_i_(τ_2_), and s_i_(τ_3_) at 0°, 90°, 180°, and 270° of the phase are taken each period T = 1/f_m_. A pixel's phase shift ϕ_i_, amplitude a_i_ and intensity b_i_, that is, the background light can be calculated by the following equations:

(1) $$\phi_{i}=\tan^{-1}\left[\frac{{\text{s}}_{\text{i}}(\tau_{0})-{\text{s}}_{\text{i}}(\tau_{2})}{{\text{s}}_{\text{i}}(\tau_{1})-{\text{s}}_{\text{i}}(\tau_{3})}\right]$$

(2) $${\text{a}}_{\text{i}}=\frac{\sqrt{\left[{\text{s}}_{\text{i}}(\tau_{0})-{\text{s}}_{\text{i}}(\tau_{2})]-[{\text{s}}_{\text{i}}(\tau_{1})-{\text{s}}_{\text{i}}(\tau_{3})\right]}}{2}$$

(3) $${\text{b}}_{\text{i}}=\frac{\sum_{\text{j}=0}^{3}{\text{s}}_{\text{i}}(\tau_{\text{j}})}{4}$$

The distance measurement D = {d_i_|i = 1,…,n} between image array and object is then determined by:

(4) $${\text{d}}_{\text{i}}=\frac{\lambda_{\text{m}}}{2}\cdot \frac{\phi_{\text{i}}}{2\pi }$$

where λ_m_ is the wavelength of the modulation signal. Due to the periodicity of the modulation signal, ToF cameras have a range unambiguous of d_n_ = c/2 · f_mod_. Within this range, the distance can be calculated exclusively [32]. The range depends on the modulation frequency of the camera which defines the wavelength of the emitted signal. To compute the distances, the camera evaluates the phase shift between a reference signal and the received signal. ϕ_i_ is proportional to the distance d.

Derawi *et al.* used ToF systems for human gait recognition by extracting gait features from the different joints and segments of the body [41].

In a recent study, Samson *et al.* used a ToF camera to analyse dynamic footprint pressures with high resolution [42].

#### 3.1.3. Structured Light

Structured light is the projection of a light pattern (beam, plane, grid, coded light, *etc.*) under geometric calibration on an object whose shape is to be recovered. The illumination pattern captured varies depending on the beam used: single dot, slit or grating stripes pattern. In these techniques, three-dimensional information is obtained by analysing the deformation of the projection of the pattern onto the scene with respect to the original projected pattern. 2D structured illumination is generated by a special projector or a light source modulated by a spatial light modulator [43,44]. One of the most common devices which use this technology is the Kinect sensor, which was used in [37] to create a marker-based real-time biofeedback system for gait retraining. In [36], stride durations and arm angular velocities were calculated using a markerless system with a Kinect sensor.

#### 3.1.4. Infrared Thermography (IRT)

**Figure 3.**
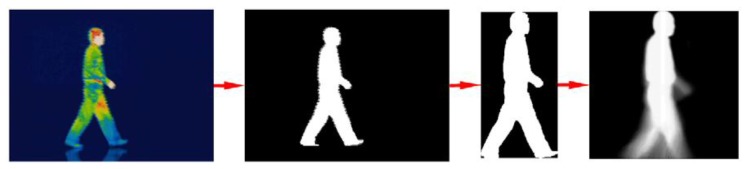
IRT image processing to extract the essential gait features. Reproduced with permission from Xue *et al.* [38].

ITG is the process of creating visual images based on surface temperatures. The ability to accurately measure the infrared thermal intensity of the human body is made possible because of the skin's emissivity is 0.98 ± 0.01, which is independent of pigmentation, absorptivity (0.98 ± 0.01) reflectivity (0.02) and transmissivity (0.000) [45]. This method was applied in [38] to recognize human gait patterns and achieved 78%–91% for probability of correct recognition (Figure 3).

### 3.2. Floor Sensors

**Figure 4.**
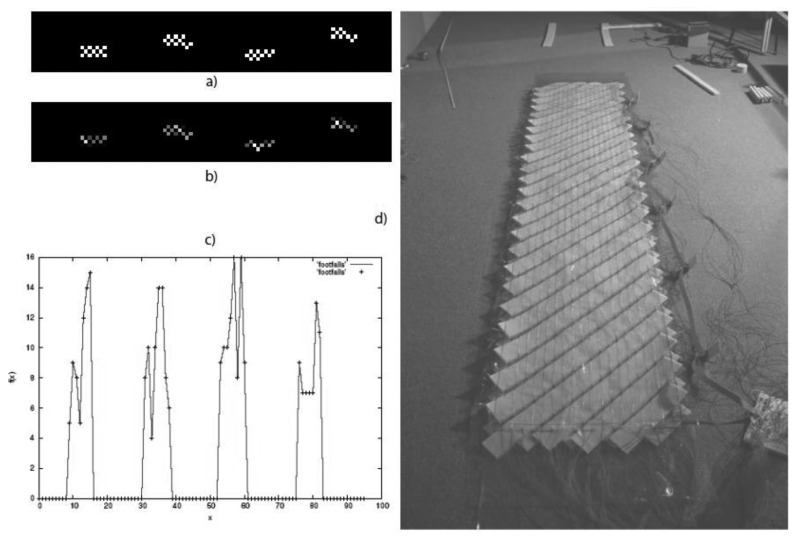
Gait analysis using floor sensors. (**a**) Steps recognized; (**b**) time elapsed in each position; (**c**) profiles for heel and toe impact; and finally (**d**) image of the prototype sensor mat on the floor. Reproduced with permission from University of Southampton.

In the systems based on this technique, sensors are place along the floor on the so called “force platforms” or instrumented walkways where gait is measured by pressure or force sensors and moment transducers when the subject walks on them. There are two types of floor sensors: force platforms and pressure measurement systems. Force platforms should be distinguished from pressure measurement systems which, although they too quantify the centre of pressure, do not directly measure the force vector applied. Pressure measurement systems are useful for quantifying the pressure patterns under a foot over time but cannot quantify horizontal or shear components of the applied forces [46]. An example of an instrumented floor sensor and the acquired data from a research conducted in University of Southampton is depicted in Figure 4.

The characteristic that distinguishes FS-based systems from IP-based systems is the analysis of force transmitted to the floor when walking, known as Ground Reaction Force (GRF). This type of system is used in many gait analysis studies [47,48]. In [49], a comparative assessment of the spatiotemporal information contained in the footstep signals for person recognition was performed analysing almost 20,000 valid footstep signals.

These devices are the most basic ones that can be used to obtain a general idea of the gait problems patients may have. Since the reaction force is exactly the opposite of the initial force (Newton's third law), the specialist finds out the evolution of the foot's pressure on the floor in real time. These data, added to the previous comparison, help specialist to make diagnoses. Pressure is given in percentage of weight in order to compare the patients’ data. Pressure varies during the time the foot is in contact with the floor. The maximum pressure occurs when the heel touches the floor and when the toes push off to take another step. During this time, pressure may reach up to 120%–150% of the patient's body weight.

**Figure 5.**
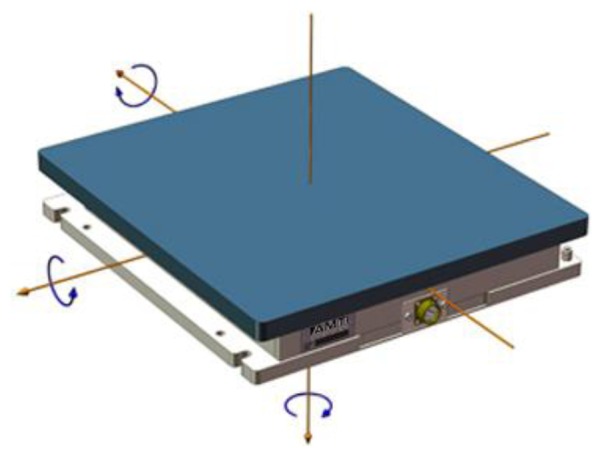
Example of AMTI Force Plate showing the three forces and the three moment components along the three measurable GFR axis. Reproduced with permission from AMTI.

The most complex systems have a sensor matrix (up to four sensors per cm^2^) which makes it possible to measure the differentiated pressure of each zone of the foot separately over time to obtain more significant information on the patient's ailment. Some examples of commercial force platforms and baropodometric mats are:

- Force platform AMTI series OR6-7 of Biometrics France (Figure 5)
- Kistler force plates of different types
- Dynamometric mat ADAL of Tecmachine
- MatScan System made by Tekscan (43.6 × 36.9 cm)
- Walking mat made by RM.Lab (150 × 50 cm)
- FootScan Plates made by RSScan.Lab (up to 200 × 40 cm)
- FDM-T System for stance and gaits analysis made by Zebris (150 × 50 cm)

### 3.3. Wearable Sensors

In gait analysis using wearable sensors, these are placed on various parts of the patient's body, such as the feet, knees or hips to measure different characteristics of the human gait. This is described in several recent reviews [50,51].This section offers a brief overview of the different types of sensors which are most commonly used in research. They include force sensors, accelerometers, gyroscopes, extensometers, inclinometers, goniometers, active markers, electromyography, *etc.*

#### 3.3.1. Pressure and Force Sensors

**Figure 6.**
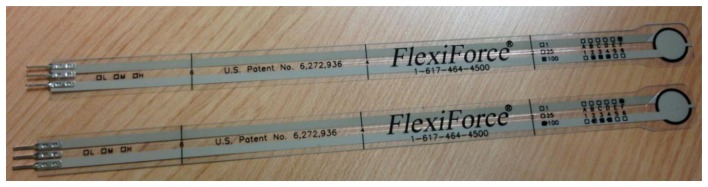
FlexiForce piezoresistive pressure sensor.

Force sensors measure the GRF under the foot and return a current or voltage proportional to the pressure measured. Pressure sensors, however, measure the force applied on the sensor without taking into account the components of this force on all the axes. The most widely used models of this type are capacitive, resistive piezoelectric and piezoresistive sensors. The choice of sensor depends on the range of pressure it will stand, linearity, sensitivity and the range of pressure it offers:

- In resistive sensors, their electrical resistance decreases as the weight placed on them increases (Figure 6).
- Piezoelectric sensors: These sensors are made of three deformation meters in three different orthogonal directions and are placed on silicone gel. Under pressure, the gel is deformed and the meters calculate this deformation. If the deformation meter and the gel characteristics are known, the total pressure can be calculated. These sensors are known for their excellent linearity and reactivity but do not adapt to surfaces due to their large size.
- Capacitive sensors: These sensors are based on the principle that the condenser capacity changes depending on different parameters, including the distance between the two electrodes.

**Figure 7.**
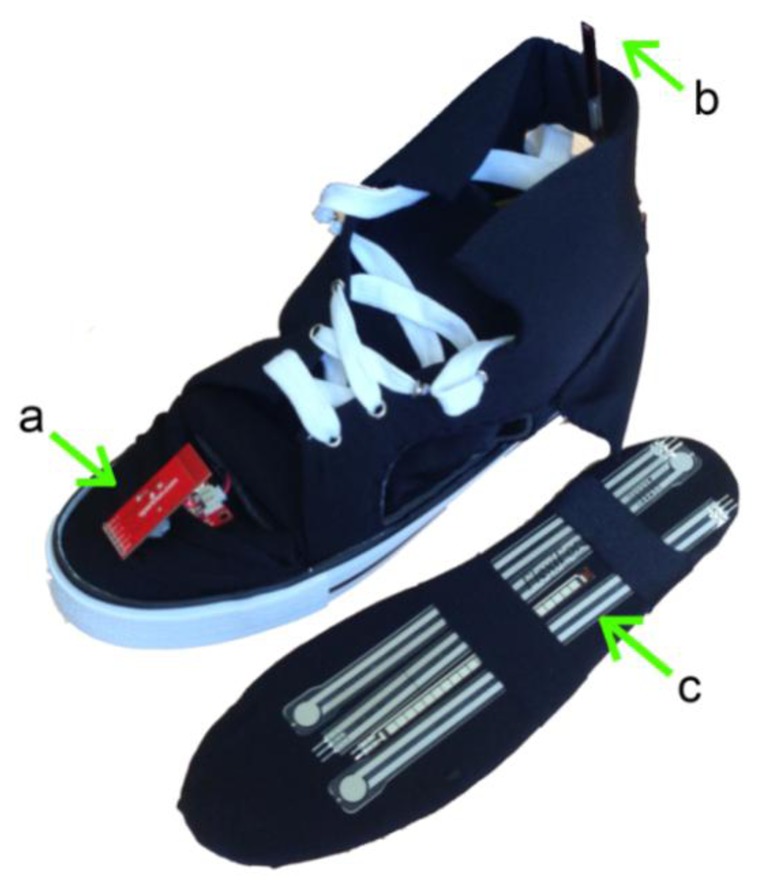
Instrumented shoe from Smartxa Project: (**a**) inertial measurement unit; (**b**) flexible goniometer; and (**c**) pressure sensors which are situated inside the insole.

This type of sensor is widely used in wearable gait analysis systems by integrating them into instrumented shoes (Figure 7) such as those developed in [52], or into baropodometric insoles [53,54]. Howell *et al.*'s study demonstrated that the GRF measurements obtained with an insole containing 12 capacitive sensors showed a high correlation to the simultaneous measurements from a clinical motion analysis laboratory [55]. Another innovative system was created by Lincoln *et al* [56], using reflected light intensity to detect the proximity of a reflective material, and was sensitive to normal and shear loads.

#### 3.3.2. Inertial Sensors

Inertial sensors are electronic devices that measure and report on an object's velocity, acceleration, orientation, and gravitational forces, using a combination of accelerometers and gyroscopes and sometimes magnetometers. An accelerometer basically uses the fundamentals of Newton's Laws of Motion, which say that the acceleration of a body is proportional to the net force acting on the body. If we know the proportionality quotient (mass of the object), and all the forces (measured with the sensors), we can calculate the acceleration. With 3-axis accelerometers and 3-axis gyroscopes, it is possible to obtain the acceleration and angular velocity. By taking the integral of the acceleration, we obtain the velocity, and by integrating the velocity, we obtain the position as refers to the 3 axes. By integrating the angular velocity, we obtain the flexion angle. Thus, analysing the signals from the accelerometers by filtering and classifying algorithms, we can extract the number of steps taken in a determined time lapse. This type of sensors may be fitted within an IMU device (Inertial Measurement Unit).

Gyroscopes are based on another property, which implies that all bodies that revolve around an axis develop rotational inertia (they resist changing their rotation speed and turn direction). A body's rotational inertia is determined by its moment of inertia, which is a rotating body's resistance to change in its rotation speed. The gyroscope must always face the same direction, being used as a reference to detect changes in direction.

Inertial Measurement Units (IMUs) are one of the most widely used types of sensors in gait analysis. Anna *et al.* developed a system with inertial sensors to quantify gait symmetry and gait normality [57], which was evaluated in-lab, against 3D kinematic measurements; and also *in situ*, against clinical assessments of hip-replacement patients, obtaining a good correlation factor between the different methods. In another recent study, Ferrari *et al.* presented an algorithm to estimate gait features which were compared with camera-based gold standard system outcomes, showing a difference in step length below 5% when considering median values [58]. In diseases where gait disorders are a symptom such as Parkinson's, we find several applications of sensors of this type [59]: Tay *et al.* presented a system with two integrated sensors located at each ankle position to track gait movements and a body sensor positioned near the cervical vertebra to monitor body posture. The system was also able to measure parameters such as maximum acceleration of the patients during standing up, and the time it takes from sit to stand [60].

**Figure 8.**
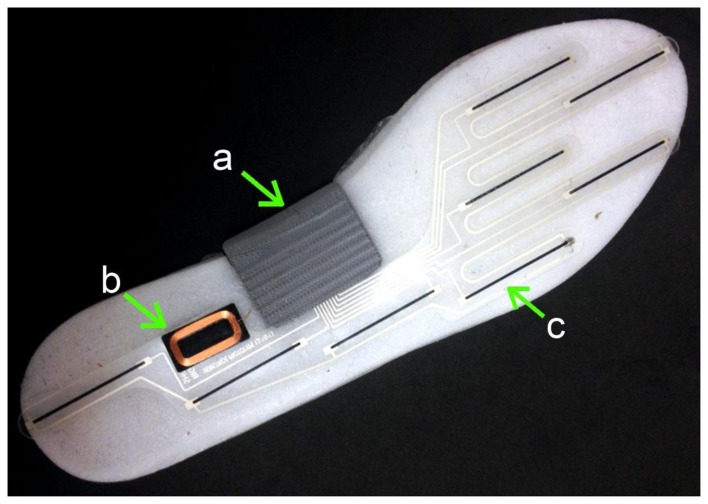
Instrumented insole: (**a**) inertial sensor, Bluetooth, microcontroller and battery module; (**b**) coil for inductive recharging; and (**c**) pressure sensors. Reproduced with permission from Stacy Morris Bamberg (Veristride, Salt Lake City, UT, USA).

The miniaturization of inertial sensors allows the possibility of integrating them on instrumented insoles for gait analysis, such as the Veristride insoles developed by Bamberg *et al.*, which additionally include specially designed pressure sensors for distributed plantar force sensing, Bluetooth communication modules and an inductive charging system (Figure 8).

#### 3.3.3. Goniometers

**Figure 9.**
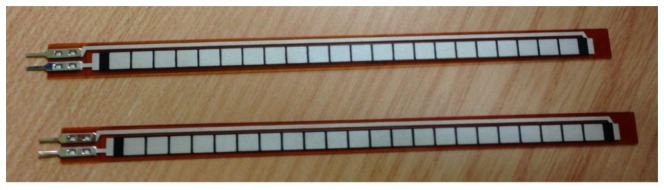
Flexible Goniometer.

These sensors can be used to study the angles for ankles, knees, hips and metatarsals. Strain gauge-based goniometers (Figure 9) work with resistance that changes depending on how flexed the sensor is. When flexed, the material forming it stretches, which means the current going through it has to travel a longer path. Thus, when the sensor is flexed, its resistance increases proportionally to the flex angle. Other types include the inductive or mechanical goniometers, and in their recent work, Dominguez *et al.*, developed a digital goniometer based on encoders to measure knee joint position [61]. These sensors are usually fitted in instrumented shoes to measures ankle to foot angles [62].

#### 3.3.4. Ultrasonic Sensors

As was described above, other important data to analyse are short step and stride length and the separation distance between feet. Ultrasonic sensors have been used to obtain these measurements [63,64]. Knowing the speed at which sound travels through the air, ultrasonic sensors measure the time it takes to send and receive the wave produced as it is reflected on an object. Knowing the time it takes the signal to travel and come back, and the speed, we can obtain the distance between the two points. The measurement range varies between 1.7 cm and nearly 450 cm. It is also possible to use this sensor to obtain other data such as the distance between the foot and the floor itself.

#### 3.3.5. Electromyography (EMG)

**Figure 10.**
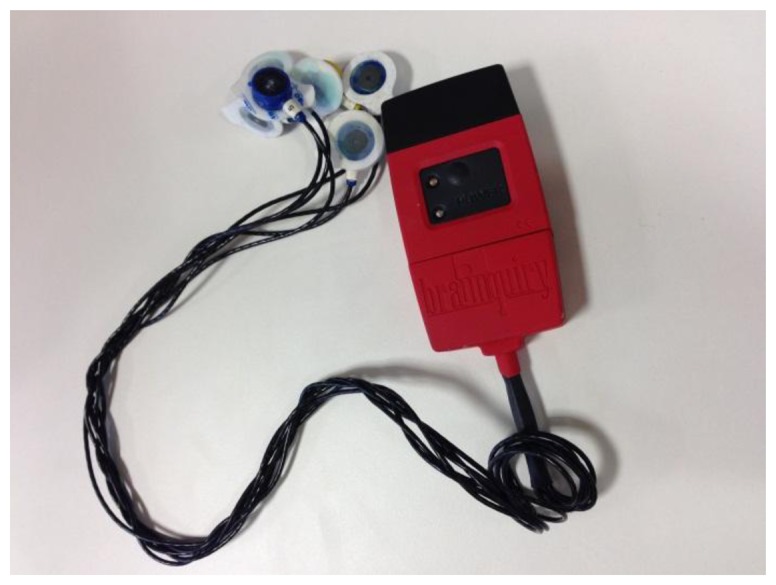
Brainquiry Wireless EMG/EEG/ECG system.

The electromyogram (EMG) is an electrical manifestation of the contracting muscle—this can be either a voluntary or involuntary muscle contraction. The EMG signal is obtained from the subject by either measuring non-invasively with surface electrodes (Figure 10), or invasively with wire or needle electrodes. The measured signal is then amplified, conditioned and recorded to yield a format that is most suitable for answering the clinical or scientific question of concern. The measurement and recording of a complex analog signal such as EMG is a complex subject as the signals of interest are invariably very small (in the order of 0.00001 to 0.005 of a Volt). It has been shown that application of surface electromyography (SEMG) is a useful in non-invasive assessment of relevant pathophysiological mechanisms potentially hindering the gait function such as changes in passive muscle-tendon properties (peripheral, non-neural component), paresis, spasticity, and loss of selectivity of motor output in functionally antagonist muscles [65]. Furthermore, EMG signals can be used to measure different gait characteristics: kinematic plots of joint angular motion can be compared to the EMG plots recorded at the same time to see if one set of data can explain the other, the amplitude of EMG signals derived during gait may be interpreted as a measure of relative muscle tension and it has been found that the EMG amplitude increases with increased walking speed and that the EMG activity is minimized with subjects walking at a comfortable speed. In a recent study performed by Wentink *et al.* [66], it was determined that EMG measured at a prosthetic leg can be used for prediction of gait initiation when the prosthetic leg is leading, predicting initial movement up to 138ms in advance in comparison to inertial sensors.

### 3.4. Commercialized Gait Analysis Systems and Laboratories

**Figure 11.**
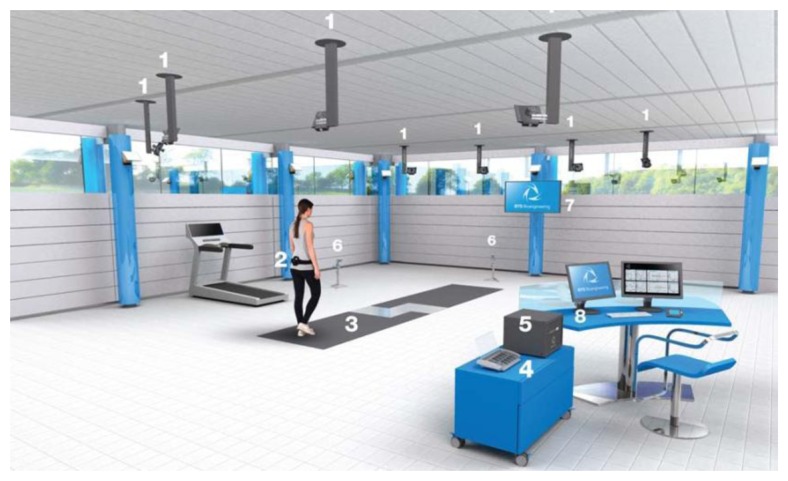
Example of NWS system: BTS GaitLab configuration. (**1**) infrared videocameras; (**2**) inertial sensor; (**3**) GRF measurement walkway; (**4**) wireless EMG; (**5**) workstation; (**6**) video recording system; (**7**) TV screen; (**8**) control station. Reproduced with permission from BTS Bioingenieering.

There are many commercial WS systems and NWS gait analysis laboratories which use different combinations of the abovementioned sensors and technologies. Some examples of NWS systems situated and calibrated in laboratory or clinical environments, such as the one depicted in Figure 11, are CONTEMPLAS: Clinical gait analysis based on a walkway [67], Tekscan: Pressure Mapping [68], GRAIL: Gait Real-time Analysis Interactive Lab, from Motek Medical [69] and BTS GAITLAB [70].

**Figure 12.**
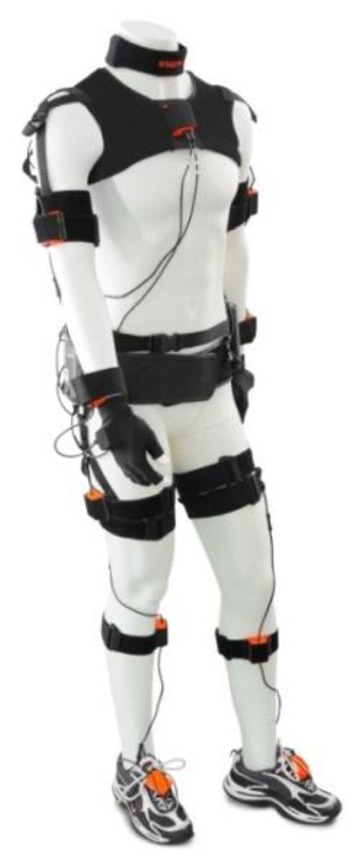
Commercial WS system based on inertial sensors: Xsens MVN. Reproduced with permission from Xsens.

Moreover, successful gait analysis systems based on wearable sensors have been commercialized, such as the widely used Xsens MVN [71], which uses 17 inertial trackers situated in the chest, upper and lower limbs to perform motion capture and six degrees of freedom tracking of the body with a wireless communicated suit (Figure 12).

**Figure 13.**
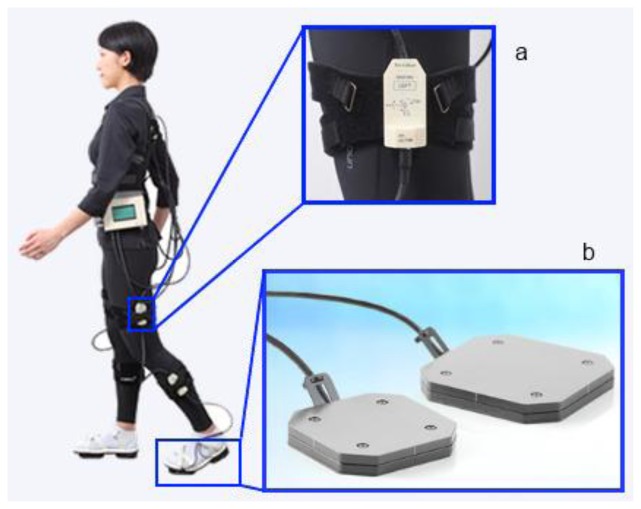
WS system based on (**a**) inertial sensors and (**b**) wearable force plates. Reproduced with permission from Tec Gihan Co.

Another commercial package is the wireless M3D gait analysis system (Figure 13) developed by Tec Gihan Co [72], which uses motion sensors on the lower leg, the thigh, the waist and the back and wearable force plates on the toes and the heels. M3D force plates measure three component forces and three moments along three orthogonal axes and include an accelerometer, a 3-axis gyroscope sensor and a 3-axis geomagnetic sensor. A similar wireless system, composed of 9 inertial sensors situated in the lower limbs and wearable force plates with wireless 6-axial force sensors, was presented by INSENCO Co. under the name Human Dynamics Analysis (HDA) [73].

## 4. Discussion

The present paper aims to provide a description of technologies and methods used for gait analysis, covering both semi-subjective and objective approaches. This section includes a discussion of the different methods. Firstly, semi-subjective and objective methods are compared. On the second and third subsections, we discuss the specific characteristics of NWS and WS systems separately, highlighting the most recent developments. Subsection 4 presents an analysis of the advantages and disadvantages of objective methods, contrasting NWS with WS. Subsection 5 offers a discussion based on the criteria that determine the various user or group profiles that benefit from gait analysis. Finally, taking into account the analysis of the limitations shown by the different models, areas for future research are put forth.

**Figure 14.**
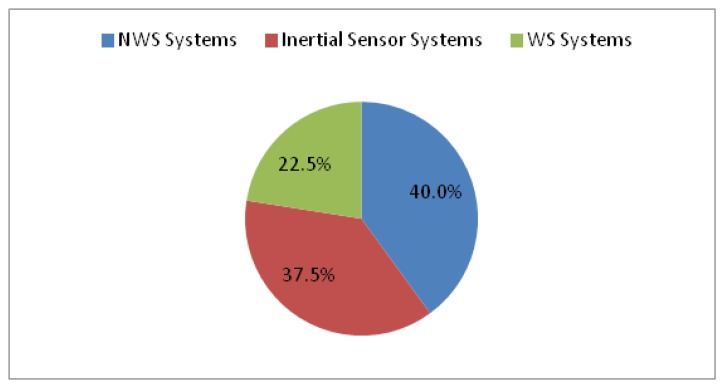
Classification of the reviewed papers published in 2012 and 2013.

Thirty two articles based on original research from 2012 and 2013 were reviewed for this paper, plus several technological and clinical reviews from the same years. 40% of these articles were related to NWS systems, 37.5% presented inertial sensor-based systems, and the remaining 22.5% corresponded to other WS systems as shown in Figure 14.

### 4.1. Comparison of Semi-Subjective and Objective Methods

In clinical conditions, gait analysis has traditionally been conducted through semi-subjective methods based on observation of patients by one or more specialists who evaluated various gait parameters. The advantage of these methods is that they do not require special equipment and only need a trained specialist to carry out the test. However, the subjective nature of the evaluation affects the accuracy, exactitude, repeatability and reproducibility of the measurements. Objective methods which use advances in technological development on sensors have appeared, with a view to more accurately quantifying the different parameters that characterise the human gait. These methods give more accurate evaluation data, making it possible to obtain information which cannot be provided by simply watching a patient walk. Examples include the GRF, the force exerted by the different muscles and angles of body segments on the different joints. A recent study [74] compared the results of one healthy subject's gait analysis results at seven different laboratories and showed that the different methods used in the various laboratories correctly measured the gait parameters. The differences found were generally lower than the established minimum detectable changes for gait kinematics and kinetics for healthy adults, thus marking promising progress in objective quantification of these parameters.

The two main approaches of these objective techniques are based on WS and NWS. It cannot be stated that one is better than the other because each one has different characteristics that make it more suitable for certain types of study.

### 4.2. Analysis of Characteristics of NWS Systems

The NWS-based methods are conducted in laboratories or controlled conditions where data retrieval devices such as cameras, laser sensors or ToF, pressure platforms or mats have been placed and set to measure gait variables as the subject walks on a clearly defined walkway. The advantage of these systems is that they isolate the study from external factors which could affect the measurements, thus allowing a more controlled analysis of the parameters being studied and obtaining high repeatability and reproducibility levels.

One of the NWS methods that shows promising results and is increasingly being used is the ToF, due to its characteristics in comparison to other image depth measurement systems. One of the newest applications of this technology is its use in higher resolution calculation of pressure in comparison with the 4 sensors/cm^2^ pressure measurement systems, as demonstrated recently by Samson *et al.* [42].

Table 2 shows a comparison of the different depth measurement techniques, with the mention of specific accuracy levels obtained in the literature. We can observe that ToF and Infrared Thermography demand the use of more expensive data acquisition equipment. Camera triangulation method can be performed without the need of special videocameras, but demand high computational cost due to the stereoscopic calculation algorithms needed to calculate the distance and position of the analysed subject. Structured light methods have become popular, in part due to the price and availability of the sensors in comparison to other image processing technologies.

Accuracy has been presented according to the results found in the literature. As each of the reviewed systems analysed different gait characteristics and had different objectives, the accuracy corresponds to the specific results of the reference.

NWS methods are usually more expensive due to the need to set up the measurement laboratory. However, new low-cost, portable systems which do not require that any sensors be attached to the body have been developed, such as the Kinect sensor. Recent studies have shown the validity of this device for gait analysis. Clark *et al.* [37] compared the results obtained with the Kinect sensor and those obtained with a marker-based 3D motion analysis (3DMA) system, observing that the lateral trunk lean angle data obtained from the Kinect system performing individualized calibration (P < 0.001) showed an error of only 0.8 ± 0.8°. In another recent study, Gabel *et al.* presented a gait analysis system based on the same sensor which also measured stride intervals more accurately using information from the entire body [36] thus proposing an inexpensive markerless system for continuous gait tracking at home.

A different type of NWS systems are those based on floor sensors. They can be very useful because patients can walk on them wearing shoes, barefoot or with a walking stick, according to how the patient usually walks. There is no need to carry other devices. The patient only has to walk on the device to obtain results. Analysis of the results makes it possible to know the pressure intensity and pressure time at each point. The main problem of these systems is their limited size, making it impossible to collect much data successively from the same patient. It is usually possible to take only 4 or 5 steps in a straight line. For this reason, the patient has to walk on the mat for a long time to obtain valid statistical data. Furthermore, depending on the length of the mat, the patient has to take care to place his/her feet carefully so that the device obtains an impression of the whole step. This can change the way patients normally walk, affecting the repeatability of the measurements.

The biggest disadvantage of NWS systems is that they do not allow evaluation and monitoring of the patient's gait during his/her everyday activities, thus extrapolating the conclusions from a short time of study that does not reflect the patient's real condition.

### 4.3. Analysis of Characteristics of WS Systems

In contrast to the disadvantages of NWS systems, the WS systems based on development of new miniaturised sensors and wireless communication systems such as Bluetooth or Zigbee have made it possible to obtain measurements of the different aspects of the human gait in real time by placing devices on different parts of the body to evaluate gait during the patient's everyday activities outside the laboratory. Moreover, sensors such as pressure and bend sensors, accelerometers and gyroscopes may be used with in-lab analysis to provide cheaper gait analysis systems that can be deployed anywhere. Fields like wearable gait retraining could enable benefits from laboratory retraining systems to extend to a broad portion of the population, which does not live near or have access to laboratory gait retraining testing facilities [75].

Trends clearly point to more research focusing on the development of wearable gait analysis systems, such as the instrumented insole developed by Howell *et al.* [55], who demonstrated that the insole results for ground reaction force and ankle moment highly correlated with data collected from a clinical motion analysis laboratory (all >0.95) for all subjects. Insole pressure sensors have proven to be an inexpensive accurate method to analyse the various step phases [51].

One of the most promising and widely used wearable sensors in recent studies is the inertial sensor. In the following paragraphs, we present an account of studies that demonstrate the validity and wide range of applications of this type of sensor in recent researches.

Studies such as Anna *et al.*'s [57], in which they contrast gait symmetry and gait normality measurements obtained with inertial sensors and 3D kinematic measurements and clinical assessments, demonstrate that the inertial sensor-based system not only correlates well with kinematic measurements obtained through other methods, but also corroborates various quantitative and qualitative measures of recovery and health status. This type of sensor has also proven to be very useful to create fall-risk prediction models with a high degree of accuracy (62%–100%), specificity (35%–100%) y sensitivity (55%–99%), depending on the model, as shown in the study by Howcroft *et al.* [76]. Adachi *et al.* developed a walking analysis system that calculates the ground reaction force, the pressure centre, reactions and movement of each joint and the body orientations based on portable force plates and motion sensors. They compared a 3D motion analysis system with their system and showed its validity for measurements of ground reaction force and the pressure centre [77]. Novak *et al.* have recently developed a system based on inertial and pressure sensors to predict gait initiation and termination. They demonstrated that both types of sensors allow timely and accurate detection of gait initiation, with overall good performance in subject-independent cross-validation, whereas inertial measurement units are generally superior to pressure sensors in predicting gait termination [78].

Inertial sensors can be used to estimate walking speed by various methods, which are described in the review by Yang and Li [79].With a view to improving the usability of these systems, studies such as Salarian *et al.*'s [80] focus on reducing the number of sensors that have to be placed on the body. They have also have managed to estimate movements of thighs from movements of shanks to reduce the number of sensing units needed from 4 to 2 in the context of ambulatory gait analysis.

As inertial sensors have been integrated in commercial mobile devices, a wide range of applications that use them to offer simple inexpensive gait analysis systems have appeared for use in fields such as telemedicine and telerehabilitation [81]. Cases in point include the one developed by Kashihara *et al.* [82] and Susi *et al.*'s [83] work on motion mode recognition and step detection. Given the potential of these mobile devices for widespread use, these developments make it possible to provide many people with gait analysis systems.

Moreover, novel research works have developed gait analysis systems using technologies that have not been traditionally applied in this field. For instance, a novel research conducted by Chen *et al.* [84] proposed a locomotion mode classification method based on a wearable capacitive sensing system as alternative to EMG, measuring ten channels of capacitance signals from the shank, the thigh, or both, with a classification accuracy of 93.6% on able-bodied subjects. Other research investigated the possible application of Ultra Wide Band (UWB) technologies in the field of gait analysis, such as the system developed by Qi *et al.* [85], which uses two UWB transceivers situated near the heel and toe to monitor the vertical heel/toe clearance during walking. They calculated toe-off, toe-strike, heel-strike and heel-off gait events by detecting the propagation delay from the reflected signals from the ground, and demonstrated the feasibility of the method comparing it with an ultrasound system with a correlation value of 0.96.

However, WS systems have certain disadvantages. In systems using accelerometers and gyroscopes to estimate speed and the distance travelled, there is a tendency to use the direct integration method with 2D or 3D IMUs, which leads to an amplification of the measurement error, making this one of the disadvantages of this technique. Analysis of inertial sensor signals is computationally complex and presents the problem of excessive noise. It is difficult to accurately calculate the paths and distances travelled.

A further disadvantage is the need to place devices on the subject's body, which may be uncomfortable or intrusive. In clinical conditions, accelerometers give a great deal of information. However, it is not enough to diagnose diseases such as Parkinson's or others in which gait disorders are an indicator because many balance and gait impairments observed are not specific to each disease. Nor have they been related to specific pathophysiologic biomarkers, as noted in the conclusions related to Parkinson's disease by Horak and Mancini [86]. Wireless gait analysis systems normally store information on SD cards or transmit it with technologies such as Bluetooth or Zigbee, which requires high energy consumption. The most commonly used energy sources are lithium batteries and if gait is to be monitored over a long period of time, the duration of the batteries may be a problem.

### 4.4. NWS and WS Systems: A Comparison

This section presents a comparison between the general advantages and disadvantages of NWS and WS systems taking into account different factors, such as power consumption, limitations, price and parameter measurement range (Table 3), and a more detailed comparison of the current specific techniques of each approach with a classification depending on type, application, accuracy, price and ease of use (Table 4).

Observing the results found in the literature, we can conclude that although all gait analysis methods can be used for general analytical purposes, when higher accuracy is needed in the detection and analysis of more specific parameters, it is necessary to choose the adequate method. The approaches that allow simultaneous, in-depth analysis of a higher number of parameters are the NWS systems on a laboratory environment, and more specifically those which are based in a combination of several of the described techniques, such as marker or markerless based image processing, EMG, inertial and floor sensors. However, the latest developments in WS allow cost-effective, non-intrusive methods which offer convenient solutions to specific analytical needs.

### 4.5. Collective-Oriented Gait Analysis System Classification

One of the key criteria to keep in mind when comparing the different gait analysis methods is the target user or group profile. The system chosen must accurately measure the key gait parameters for that particular group. When focusing on the clinical applications of gait analysis, the end users can be divided into the following groups: (a) patients with neurological diseases; (b) patients suffering from systemic diseases such as cardiopathies; (c) patients with stroke sequelae and (d) the elderly. Each of these groups shows different characteristics for gait-related disorders. Patients suffering from neurological diseases such as Parkinson's show short step length, shuffling gait and some patients experience freezing of gait (FoG), a sudden and unexpected inability to start or continue walking that can be responsible for falls [90]. In these cases, image-based NWS systems may offer more accurate step length results than inertial sensor-based WS systems in which the estimated step length gives an error due to double integration of accelerometer signals. In patients with cardiopathies, slow gait is one of the most common indicators among post-AMI older adults and is associated with increased all-cause readmission at one year, according to [91]. Therefore, the methods used to assess the condition of patients suffering from this type of ailment should achieve high accuracy measurements of velocity. Again, in the case of inertial sensor systems, the inertial sensor measurement error is unavoidable, especially for miniature sensors. Therefore, an appropriate method should be chosen. Stroke patients often from suffer abnormal patterns of motion which alter the velocity, length of the stride, cadence, and all phases of the gait cycle [92], especially due to decreased velocity on the hemiplegic side, which is strongly associated with the clinical severity of muscle weakness. As velocity improved, these abnormal movements decreased. For this reason, study of muscular activity through use of techniques such as EMG is especially important in these cases. Lastly, gait disorders associated with ageing-related diseases may also be due to multiple factors, as shown in detail in the work by Jahn *et al.* [93]. This study indicates that a broad approach should be taken when analysing gait characteristics in the elderly. Therefore, although minor differences exist between the appropriateness of the different methods for each target group, we cannot indicate key factors that make it possible to link each group with the most suitable method.

### 4.6. Considerations for Future Research

In view of the advantages WS systems show when measuring and evaluating the human gait, interfering as little as possible with the subject's daily activities, and in order to overcome the present limitations of gait measurement systems, future research should focus on four different areas: (1) new sensors for in-depth parameter analysis; (2) power consumption; (3) miniaturization; and (4) signal processing algorithms. Each of the areas is detailed below.

Area 1 refers to the need for new wearable sensors that make it possible to quantify a higher number of gait parameters to reach the capacity and accuracy of NWS systems. More specifically, new sensors which provide more accurate measurements of segment position/orientation and velocity, joint angles, pressure distributions, step recognition and length, among others, are needed. Work should also be done to determine the most promising sensor locations for each research purpose. On area 2, work should focus on the development of technologies allowing for greater working autonomy and extended duration of energy sources in order to carry out analyses over long time periods. Power consumption is an important limitation of the current gait analysis systems, and it interferes directly with the capacity of the system to measure and monitor the gait parameters over long time periods. Future research should intend to develop new energy supply systems with extended battery life duration, and energy-efficient gait analysis systems which need less energy to perform their functions. Emphasis should also centre on area 3, miniaturisation of the measuring and communication systems to create fully non-intrusive invisible systems, which can then be totally integrated in the outfit or in the person's body enhancing the usability of the current systems. The miniaturization of sensors would allow combining different sensor types in a single device able to measure a wider range of parameters. Finally, future research should also focus on the development and improvement of signal processing and analysis algorithms (area 4) to make it possible to classify gait disorders reliably and match the different gait parameter measurement patterns with the different diseases indicated, thus contributing to early diagnosis and monitoring of rehabilitation processes. The current movement tracking algorithms based on the application of Kalman filters and Direction Cosine Matrix (DCM) to data acquired from gyroscopes and accelerometers should be improved.

## 5. Conclusions

In the last decades, interest in obtaining in-depth knowledge of human gait mechanisms and functions has increased dramatically. Thanks to advances in measuring technologies that make it possible to analyse a greater number of gait characteristics and the development of more powerful, efficient and smaller sensors, gait analysis and evaluation have improved. In contrast to the traditional semi-subjective methods which depend on the specialist's experience, the different parameters being studied can now be objectively quantified. These new methods have great impact in various fields such as human recognition, sports, and especially in the clinical field, where objective gait analysis plays an important role in diagnosis, prevention and monitoring of neurological, cardiopathic and age-related disorders.

This article presents a general review of the different gait analysis methods. A series of parameters have been extracted from the description of the key human gait parameters, of which we highlight the time-space group due to its importance from the clinical point of view. These parameters include walking speed, stride and step length, swing and stance times, *etc.* force-related parameters such as GRF, muscle force and joint momentum. Special importance is given to the parameters measured and monitored over long periods of time such as distance travelled and autonomy regarding number and duration of the stops, which can only be measured during daily activities using wearable sensors. Commonly used semi-subjective techniques such as TUG and Timed 25-foot Walk were then analysed due to their widespread application. We can conclude that the objective techniques classified as image processing, floor sensors and wearable sensors have characteristics that make them efficient and effective for different types of needs. The latest research on gait analysis comparing the advantages and disadvantages of the different systems leads us to conclude that, although objective quantification of the different parameters is rigorously carried out, these studies do not cover the need to extend the measurement capacity of WS systems in order to provide gait information obtained during users’ daily activities over long time periods. For this reason, areas for future research focused on the development of specific pathology-oriented systems aimed at prevention and evolution monitoring are proposed.

## Figures and Tables

**Table 1.** Overview of gait parameters and applications.

**Gait Parameter**	**Application**		

	**Clinical**	**Sports**	**Recognition**
Stride velocity	X	X	X
Step length	X	X	X
Stride length	X	X	X
Cadence	X	X	X
Step Width	X	X	X
Step Angle	X	X	X
Step time	X		
Swing time	X		
Stance time	X		
Traversed distance	X	X	
Gait autonomy	X		
Stop duration	X		
Existence of tremors	X		
Fall	X		
Accumulated altitude	X	X	
Route	X	X	
Gait phases	X	X	X
Body segment orientation	X	X	
Ground Reaction Forces	X	X	
Joint angles	X	X	
Muscle force	X	X	
Momentum	X	X	
Body posture (inclination, symmetry)	X	X	X
Long-term monitoring of gait	X	X	

**Table 2.** Characteristics of different depth measurement methods.

**Method**	**Advantages**	**Disadvantages**	**Sensor Price (€)**	**Ref.**	**Accuracy**
Camera Triangulation	- High image resolution - No special conditions in terms of scene illumination	- At least two cameras needed - High computational cost	400 to 1,900	[11,39]	70% [39]
Time of Flight	- Only one camera is needed - It is not necessary to calculate depth manually - Real-time 3D acquisition - Reduced dependence on scene illumination	- Low resolutions - Aliasing effect - Problems with reflective surfaces	239 to 3,700	[41]	2.66% to 9.25% (EER) [41]
Structured Light	- Provide great detail - Allows robust and precise acquisition of objects with arbitrary geometry and a with a wide range of materials - Geometry and texture can be obtained with the same camera	- Irregular functioning with motion scenes - Problems with transparent and reflective surfaces - Superposition of the light pattern with reflections	160 to 200	[36,37]	<1% (Mean diff) [37]
Infrared Thermography	- Fast, reliable & accurate output - A large surface area can be scanned in no time - Requires very little skill for monitoring	- Cost of instrument is relatively high - Unable to detect the inside temperature if the medium is separated by glass/polythene - Emissivity problems	1.000 to 18.440	[38]	78%–91%

**Table 3.** Comparison between NWS and WS systems.

**System**	**Advantages**	**Disadvantages**
**NWS**	- Allows simultaneous analysis of multiple gait parameters captured from different approaches - Non restricted by power consumption - Some systems are totally non-intrusive in terms of attaching sensors to the body - Complex analysis systems allow more precision and have more measurement capacity - Better repeatability, reproducibility and less external factor interference due to controlled environment. - Measurement process controlled in real time by the specialist.	- Normal subject gait can be altered due to walking space restrictions required by the measurement system - Expensive equipment and tests - Impossible to monitor real life gait outside the instrumented environment
**WS**	- Transparent analysis and monitoring of gait during daily activities and on the long term - Cheaper systems - Allows the possibility of deployment in any place, not needing controlled environments - Increasing availability of varied miniaturized sensors - Wireless systems enhance usability - In clinical gait analysis, promotes autonomy and active role of patients	- Power consumption restrictions due to limited battery duration - Complex algorithms needed to estimate parameters from inertial sensors - Allows analysis of limited number of gait parameters - Susceptible to noise and interference of external factors not controlled by specialist

**Table 4.** Classification of existing gait analysis systems.

		**Method**	**Ref.**	**Application**	**Accuracy**	**Price (€)**	**Ease of Use**
**Wearable Sensors**		Inertial sensors	[57–60,71,72,73, 76,77,78,79,82]	Segment position Step Detection Stride length	Angle Coeff. Mult. Corr. > 0.96 [71] <%5 for median values Stride length error −0.8 ± 6.6 [80]	91.30 [87]	Complex algorithms. Sensible to interferences
		GRF plates	[53–54,56, 72,73]	Step Detection GRF Gait Phase Detection	10% of the range of GRF [56]	17,180 for one foot [72]	Bigger size than pressure sensors (less usability) Easy to analyse data
		Pressure sensors	[51,52,55]	Foot Plantar Pressure Distribution Gait Phase Detection Step Detection	Pressure correlation R > 0.95 (with clinical motion analysis laboratory measures)	14.58 [87]	Simple algorithms. Easy to setup in shoe/insole. Highly nonlinear response
		EMG	[65]	Muscle Electrical Activity Gait Phase Detection	SNR = 0.25 microvolt @ 200 Hz [Brainquiry]	35–350 [88]	Need specific knowledge on electrode setup. Sensible to interferences
		UWB	[85]	Step Detection Gait Phase Detection	Correlation R = 0.96 (with ultrasound system measures) [85]	Not specified	Measurement situation on shoe/foot is critical
		Ultrasound	[63,64]	Step Length Gait Phase Detection	Not Specified	20.44 [89]	Sensible to interferences. Sensor situation is critical
		Goniometer	[61,62]	Joint Angles Step Detection	R = 0.999 with measures taken with mechanical Goniometer [61]	9.46 [87]	Easy to setup and analyse data, but low hysteresis.
**Non Wearable Sensors**	**Floor Sensors**	GRF plates	AMTI, Kistler	Step Detection GRF Gait Phase detection	±0.1% of load [AMTI]	30,000 [AMTI]	Need for the subject to contact center of plate for correct measurement
		Pressure sensor mats and platforms	[47–49]	Plantar Pressure Distribution Gait Phase detection Step Detection Gait Recognition	80% recognition rate [47] 2.5 to 10% EER in recognition [49] 72% step detection rate [48]	4,000–54,000 [depending on number of sensors and specifications]	Limitations of space, indoor measurement, and patients ability to make contact with the platform
	**Image Processing**	Single camera image processing	[27–32]	Individual Recognition Segment Position	77.8% recognition rate [27]	400–1,900 [depending on camera specifications]	Simple equipment setup. Complex analysis algorithms
		Time of Flight	[41,42]	Segment Position Gait Phase Detection Foot Plantar Pressure Distribution Individual Recognition	2.66%–9.25% EER recognition [41]	200– 3,700 [depending on sensor specifications]	Only one camera neededProblems with reflective surfaces
		Stereoscopic Vision	[11,39]	Gait Phase Detection Segment position Individual Recognition	70.18% recognition rate [39]	200–9,000 [depending on camera specifications]	Complex calibration. High computational cost
		Structured Light	[36,37]	Segment Position Gait Phase Detection	Correlation R=0.89 with inertial and pressure sensor measures [36] Angle measurement error = −0.8 ± 0.8° [37]	160–200 [depending on sensor specifications]	Complex calibration. Lower sensor cost related with other image processing systems
		IR Thermography	[38]	Gait Phase Detection Segment position Individual Recognition	78%–91% recognition [38]	8,000 to 100,000 [8 camera laboratory as BTS Gaitlab]	Need to take into account emissivity, absorptivity, reflectivity, transmissivity of materials

## References

[1] Gouwanda D., Senanayake S.M.N.A., Osman N.A.A., Ibrahim F., Abas W.A.B.W., Rahman H.S.A., Ting H.N. (2008). Emerging Trends of Body-Mounted Sensors in Sports and Human Gait Analysis. 4th Kuala Lumpur International Conference on Biomedical Engineering 2008.

[2] Di Stasi S.L., Logerstedt D., Gardinier E.S., Snyder-Mackler L. (2013). Gait patterns differ between ACL-reconstructed athletes who pass return-to-sport criteria and those who fail. Am. J. Sports Med..

[3] Lee H., Sullivan S.J., Schneiders A.G. (2013). The use of the dual-task paradigm in detecting gait performance deficits following a sports-related concussion: A systematic review and meta-analysis. J. Sci. Med. Sport.

[4] Fathima S.M.H.S.S., Banu R.S.D.W. Human Gait Recognition Based on Motion Analysis Including Ankle to Foot Angle Measurement.

[5] Wang L., Tan T., Ning H.Z., Hu W.M. (2003). Silhouette analysis-based gait recognition for human identification. IEEE Trans. Pattern Anal. Mach. Intell..

[6] Han J., Bhanu B. (2006). Individual recognition using Gait Energy Image. IEEE Trans. Pattern Anal. Mach. Intell..

[7] Derawi M.O., Bours P., Holien K. Improved Cycle Detection for Accelerometer Based Gait Authentication.

[8] Sutherland D.H. (2001). The evolution of clinical gait analysis part I: Kinesiological EMG. Gait Posture.

[9] Sutherland D.H. (2002). The evolution of clinical gait analysis. Part II kinematics. Gait Posture.

[10] Sutherland D.H. (2005). The evolution of clinical gait analysis part III—kinetics and energy assessment. Gait Posture.

[11] Gomatam A.N.M., Sasi S. (2004). Multimodal gait recognition based on stereo vision and 3D template matching. CISST.

[12] White S.C., Winter D.A. (1992). Predicting muscle forces in gait from EMG signals and musculotendon kinematics. J. Electromyogr. Kinesiol..

[13] Mummolo C., Mangialardi L., Kim J.H. (2013). Quantifying dynamic characteristics of human walking for comprehensive gait cycle. J. Biomech. Eng..

[14] Kerrigan D.C., Todd M.K., Della Croce U., Lipsitz L.A., Collins J.J. (1998). Biomechanical gait alterations independent of speed in the healthy elderly: Evidence for specific limiting impairments. Arch. Phys. Med. Rehabil..

[15] Stolze H., Klebe S., Petersen G., Raethjen J., Wenzelburger R., Witt K., Deuschl G. (2002). Typical features of cerebellar ataxic gait. J. Neurol. Neurosurg. Psychiatry.

[16] Gehlsen G., Beekman K., Assmann N., Winant D., Seidle M., Carter A. (1986). Gait characteristics in multiple sclerosis: progressive changes and effects of exercise on parameters. Arch. Phys. Med. Rehabil..

[17] Waters D.L., Hale L., Grant A.M., Herbison P., Goulding A. (2010). Osteoporosis and gait and balance disturbances in older sarcopenic obese New Zealanders. Osteoporos. Int..

[18] Arana-Arri E., Gutiérrez-Ibarluzea I., Ecenarro Mugaguren A., Asua Batarrita J. (2007). Prevalence of certain osteoporosis-determining habits among post menopausal women in the Basque Country, Spain, in 2003 (in Spanish). Rev. Esp. Salud Pública.

[19] Afilalo J., Eisenberg M.J., Morin J.F., Bergman H., Monette J., Noiseux N., Perrault L.P., Alexander K.P., Langlois Y., Dendukuri N. (2010). Gait speed as an incremental predictor of mortality and major morbidity in elderly patients undergoing cardiac surgery. J. Am. Coll. Cardiol..

[20] Cutter G.R., Baier M.L., Rudick R.A., Cookfair D.L., Fischer J.S., Petkau J., Syndulko K., Weinshenker B.G., Antel J.P., Confavreux C. (1999). Development of a multiple sclerosis functional composite as a clinical trial outcome measure. Brain.

[21] Hobart J.C., Riazi A., Lamping D.L., Fitzpatrick R., Thompson A.J. (2003). Measuring the impact of MS on walking ability: The 12-Item MS Walking Scale (MSWS-12). Neurology.

[22] Holland A., O’Connor R.J., Thompson A.J., Playford E.D., Hobart J.C. (2006). Talking the talk on walking the walk: A 12-item generic walking scale suitable for neurological conditions. J. Neurol..

[23] Tinetti M.E. (1986). Performance-oriented assessment of mobility problems in elderly patients. J. Am. Geriatr. Soc..

[24] Mathias S., Nayak U.S., Isaacs B. (1986). Balance in elderly patients: The “get-up and go” test. Arch. Phys. Med. Rehabil..

[25] Wolfson L., Whipple R., Amerman P., Tobin J.N. (1990). Gait assessment in the elderly: A gait abnormality rating scale and its relation to falls. J. Gerontol..

[26] Fried A.V., Cwikel J., Ring H., Galinsky D. (1990). ELGAM-extra-laboratory gait assessment method: Identification of risk factors for falls among the elderly at home. Int. Disabil. Stud..

[27] Pratheepan Y., Condell J.V., Prasad G. The Use of Dynamic and Static Characteristics of Gait for Individual Identification.

[28] Kusakunniran W., Wu Q., Zhang J., Li H. Support Vector Regression for Multi-View Gait Recognition Based on Local Motion Feature Selection.

[29] Chang P.C., Tien M.C., Wu J.L., Hu C.S. Real-Time Gender Classification from Human Gait for Arbitrary View Angles.

[30] Arias-Enriquez O., Chacon-Murguia M.I., Sandoval-Rodriguez R. Kinematic Analysis of Gait Cycle Using a Fuzzy System for Medical Diagnosis.

[31] Iwashita Y., Kurazume R., Ogawara K. Expanding Gait Identification Methods from Straight to Curved Trajectories.

[32] Muramatsu D., Shiraishi A., Makihara Y., Yagi Y. Arbitrary View Transformation Model for Gait Person Authentication.

[33] Jain R.C., Kasturi R., Schunck B.G. (1995). Machine Vision.

[34] Phan Ba R., Pierard S., Moonen G., van Droogenbroeck M., Belachew S. Detection and Quantification of Efficiency and Quality of Gait Impairment in Multiple Sclerosis through Foot Path Analysis. http://orbi.ulg.ac.be/handle/2268/132779.

[35] Jensen R.R., Paulsen R.R., Larsen R., Salberg A.B., Hardeberg J.Y., Jenssen R. (2009). Analyzing Gait Using a Time-of-Flight Camera. Image Analysis.

[36] Gabel M., Gilad-Bachrach R., Renshaw E., Schuster A. Full Body Gait Analysis with Kinect.

[37] Clark R.A., Pua Y.H., Bryant A.L., Hunt M.A. (2013). Validity of the Microsoft Kinect for providing lateral trunk lean feedback during gait retraining. Gait Posture.

[38] Xue Z., Ming D., Song W., Wan B., Jin S. (2010). Infrared gait recognition based on wavelet transform and support vector machine. Pattern Recognit..

[39] Liu H., Cao Y., Wang Z. Automatic Gait Recognition from a Distance.

[40] Kolb A., Barth E., Koch R., Larsen R. (2009). Time-of-Flight Sensors in Computer Graphics; EUROGRAPHICS STAR Report.

[41] Derawi M.O., Ali H., Cheikh F.A. Gait Recognition Using Time-of-Flight Sensor. http://subs.emis.de/LNI/Proceedings/Proceedings191/187.pdf.

[42] Samson W., van Hamme A., Sanchez S., Chèze L., van Sint Jan S., Feipel V. (2012). Dynamic footprint analysis by time-of-flight camera. Comput. Methods Biomech. Biomed. Engin..

[43] Geng J. (2011). Structured-light 3D surface imaging: A tutorial. Adv. Opt. Photon..

[44] Young A. (1994). Handbook of Pattern Recognition and Image Processing.

[45] Dziuban E. Human Body Temperature Measurement—Class Program. http://www.imeko.org/publications/tc1-2002/IMEKO-TC1-2002-005.pdf.

[46] Robertson G., Kamen G., Caldwell G., Hamill J., Whittlesey S. Research Methods in Biomechanics.

[47] Middleton L., Buss A.A., Bazin A., Nixon M.S. A Floor Sensor System for Gait Recognition.

[48] Leusmann P., Mollering C., Klack L., Kasugai K., Ziefle M., Rumpe B. Your Floor Knows Where You Are: Sensing and Acquisition of Movement Data.

[49] Vera-Rodriguez R., Mason J.S.D., Fierrez J., Ortega-Garcia J. (2013). Comparative analysis and fusion of spatiotemporal information for footstep recognition. IEEE Trans. Pattern Anal. Mach. Intell..

[50] Tao W., Liu T., Zheng R., Feng H. (2012). Gait analysis using wearable sensors. Sensors.

[51] Abdul Razak A.H., Zayegh A., Begg R.K., Wahab Y. (2012). Foot plantar pressure measurement system: A review. Sensors.

[52] Bae J., Tomizuka M. (2013). A tele-monitoring system for gait rehabilitation with an inertial measurement unit and a shoe-type ground reaction force sensor. Mechatronics.

[53] Savelberg H.H.C.M., Lange A.L.H.D. (1999). Assessment of the horizontal, fore-aft component of the ground reaction force from insole pressure patterns by using artificial neural networks. Clin. Biomech..

[54] Forner Cordero A., Koopman H.J.F.M., van der Helm F.C.T. (2004). Use of pressure insoles to calculate the complete ground reaction forces. J. Biomech..

[55] Howell A.M., Kobayashi T., Hayes H.A., Foreman K.B., Bamberg S.J.M. (2013). Kinetic gait analysis using a low-cost insole. IEEE Trans. Biomed. Eng..

[56] Lincoln L.S., Bamberg S.J.M., Parsons E., Salisbury C., Wheeler J. An Elastomeric Insole for 3-Axis Ground Reaction Force Measurement.

[57] Anna A.S., Wickström N., Eklund H., Zügner R., Tranberg R., Gabriel J., Schier J., Huffel S.V., Conchon E., Correia C., Fred A., Gamboa H. (2013). Assessment of Gait Symmetry and Gait Normality Using Inertial Sensors: In-Lab and In-Situ Evaluation. Biomedical Engineering Systems and Technologies.

[58] Ferrari A., Rocchi L., van den Noort J., Harlaar J., Pons J.L., Torricelli D., Pajaro M. (2013). Toward the Use of Wearable Inertial Sensors to Train Gait in Subjects with Movement Disorders. Converging Clinical and Engineering Research on Neurorehabilitation.

[59] Salarian A., Russmann H., Vingerhoets F.J.G., Dehollaini C., Blanc Y., Burkhard P.R., Aminian K. (2004). Gait assessment in Parkinson's disease: Toward an ambulatory system for long-term monitoring. IEEE Trans. Biomed. Eng..

[60] Tay A., Yen S.C., Li J.Z., Lee W.W., Yogaprakash K., Chung C., Liew S., David B., Au W.L. Real-Time Gait Monitoring for Parkinson Disease.

[61] Dominguez G., Cardiel E., Arias S., Rogeli P. A Digital Goniometer Based on Encoders for Measuring Knee-Joint Position in an Orthosis.

[62] Bamberg S., Benbasat A.Y., Scarborough D.M., Krebs D.E., Paradiso J.A. (2008). Gait analysis using a shoe-integrated wireless sensor system. Trans. Inf. Tech. Biomed..

[63] Wahab Y., Bakar N.A. Gait Analysis Measurement for Sport Application Based on Ultrasonic System.

[64] Maki H., Ogawa H., Yonezawa Y., Hahn A.W., Caldwell W.M. (2012). A new ultrasonic stride length measuring system. Biomed. Sci. Instrum..

[65] Frigo C., Crenna P. (2009). Multichannel SEMG in clinical gait analysis: A review and state-of-the-art. Clin. Biomech..

[66] Wentink E.C., Schut V.G.H., Prinsen E.C., Rietman J.S., Veltink P.H. (2014). Detection of the onset of gait initiation using kinematic sensors and EMG in transfemoral amputees. Gait Posture.

[67] Templo Clinical Gait Analysis. http://www.contemplas.com/clinical_gait_analysis_walkway.aspx.

[68] Enhance Gait Analysis with Pressure Mapping. http://www.tekscan.com/medical/gait-analysis.html?utm_source=google&utm_medium=cpc&utm_term=gait+analysis&utm_content=ad1&utm_campaign=medical&gclid=CPvH8uWjgrsCFevjwgodqFIAsQ.

[69] Grail—Gait Real-time Analysis Interactive Lab. http://www.motekmedical.com/products/grail-gait-real-time-analysis-interactive-lab/.

[70] BTS Bioengineering. http://www.btsbioengineering.com/products/integrated-solutions/bts-gaitlab/.

[71] Zhang J.T., Novak A.C., Brouwer B., Li Q. (2013). Concurrent validation of Xsens MVN measurement of lower limb joint angular kinematics. Physiolog. Meas..

[72] Tec Gihan Co., Ltd. http://www.tecgihan.co.jp/english/p7.htm.

[73] Intelligent Sensor and Control System Co., Ltd. http://www.insenco-j.com/_d275212500.htm.

[74] Benedetti M.G., Merlo A., Leardini A. (2013). Inter-laboratory consistency of gait analysis measurements. Gait Posture.

[75] Shull P.B., Jirattigalachote W., Zhu X., Lee J., Lee M.C., Liu H., Ryu J.H. (2013). An Overview of Wearable Sensing and Wearable Feedback for Gait Retraining. Intelligent Robotics and Applications.

[76] Howcroft J., Kofman J., Lemaire E.D. (2013). Review of fall risk assessment in geriatric populations using inertial sensors. J. Neuroeng. Rehabil..

[77] Adachi W., Tsujiuchi N., Koizumi T., Shiojima K., Tsuchiya Y., Inoue Y. Calculation of Joint Reaction Force and Joint Moments Using by Wearable Walking Analysis System.

[78] Novak D., Reberšek P., de Rossi S.M.M., Donati M., Podobnik J., Beravs T., Lenzi T., Vitiello N., Carrozza M.C., Munih M. (2013). Automated detection of gait initiation and termination using wearable sensors. Med. Eng. Phys..

[79] Yang S., Li Q. (2012). Inertial sensor-based methods in walking speed estimation: A systematic review. Sensors.

[80] Salarian A., Burkhard P.R., Vingerhoets F.J.G., Jolles B.M., Aminian K.A. (2013). Novel approach to reducing number of sensing units for wearable gait analysis systems. IEEE Trans. Biomed. Eng..

[81] McGuire M.L. An Overview of Gait Analysis and Step Detection in Mobile Computing Devices.

[82] Kashihara H., Shimizu H., Houchi H., Yoshimi M., Yoshinaga T., Irie H.A. Real-Time Gait Improvement Tool Using a Smartphone.

[83] Susi M., Renaudin V., Lachapelle G. (2013). Motion mode recognition and step detection algorithms for mobile phone users. Sensors.

[84] Chen B., Zheng E., Fan X., Liang T., Wang Q., Wei K., Wang L. (2013). Locomotion mode classification using a wearable capacitive sensing system. IEEE Trans. Neural Syst. Rehabil. Eng..

[85] Qi Y., Soh C.B., Gunawan E., Low K.S., Maskooki A. Using Wearable UWB Radios to Measure Foot Clearance During Walking.

[86] Horak F.B., Mancini M. (2013). Objective biomarkers of balance and gait for parkinson's disease using body-worn sensors. Mov. Disord..

[87] New Product Friday: The Keypad to my Heart. https://www.sparkfun.com/.

[88] Shimmer. http://www.shimmersensing.com/.

[89] Devantech. http://www.robot-electronics.co.uk/.

[90] Maillet A., Pollak P., Debû B. (2012). Imaging gait disorders in Parkinsonism: A review. J. Neurol. Neurosurg. Psychiatry.

[91] Dodson J.A., Reid K.J., Gill T.M., Krumholz H.M., Forman D.E., Spertus J.A., Rich M.W., Arnold S.V., Alexander K.P. (2012). Slow gait among older adults post-ami and risk for hospital readmission. J. Am. Coll. Cardiol..

[92] De Quervain I.A., Simon S.R., Leurgans S., Pease W.S., McAllister D. (1996). Gait pattern in the early recovery period after stroke. J. Bone Joint Surg. Am..

[93] Jahn K., Zwergal A., Schniepp R. (2010). Gait disturbances in old age (in German). Dtsch. Ärztebl. Int..

